# Single Cell Transcriptomics Implicate Novel Monocyte and T Cell Immune Dysregulation in Sarcoidosis

**DOI:** 10.3389/fimmu.2020.567342

**Published:** 2020-12-08

**Authors:** Lori Garman, Richard C. Pelikan, Astrid Rasmussen, Caleb A. Lareau, Kathryn A. Savoy, Umesh S. Deshmukh, Harini Bagavant, Albert M. Levin, Salim Daouk, Wonder P. Drake, Courtney G. Montgomery

**Affiliations:** ^1^ Oklahoma Medical Research Foundation, Genes and Human Disease, Oklahoma City, OK, United States; ^2^ Cell Circuits and Epigenomics Program, Broad Institute, Cambridge, MA, United States; ^3^ Oklahoma Medical Research Foundation, Arthritis and Clinical Immunology, Oklahoma City, OK, United States; ^4^ Department of Public Health Sciences, Henry Ford Health System, Detroit, MI, United States; ^5^ Cardiovascular Institute, University of Oklahoma Health Sciences Center, Oklahoma City, OK, United States; ^6^ Vanderbilt University School of Medicine, Nashville, TN, United States

**Keywords:** sarcoidosis, regulatory T cells, classical monocytes, RNA sequencing analysis, lymphocyte activation, cell migration

## Abstract

Sarcoidosis is a systemic inflammatory disease characterized by infiltration of immune cells into granulomas. Previous gene expression studies using heterogeneous cell mixtures lack insight into cell-type-specific immune dysregulation. We performed the first single-cell RNA-sequencing study of sarcoidosis in peripheral immune cells in 48 patients and controls. Following unbiased clustering, differentially expressed genes were identified for 18 cell types and bioinformatically assessed for function and pathway enrichment. Our results reveal persistent activation of circulating classical monocytes with subsequent upregulation of trafficking molecules. Specifically, classical monocytes upregulated distinct markers of activation including adhesion molecules, pattern recognition receptors, and chemokine receptors, as well as enrichment of immunoregulatory pathways HMGB1, mTOR, and ephrin receptor signaling. Predictive modeling implicated TGFβ and mTOR signaling as drivers of persistent monocyte activation. Additionally, sarcoidosis T cell subsets displayed patterns of dysregulation. CD4 naïve T cells were enriched for markers of apoptosis and Th17/T_reg_ differentiation, while effector T cells showed enrichment of anergy-related pathways. Differentially expressed genes in regulatory T cells suggested dysfunctional p53, cell death, and TNFR2 signaling. Using more sensitive technology and more precise units of measure, we identify cell-type specific, novel inflammatory and regulatory pathways. Based on our findings, we suggest a novel model involving four convergent arms of dysregulation: persistent hyperactivation of innate and adaptive immunity *via* classical monocytes and CD4 naïve T cells, regulatory T cell dysfunction, and effector T cell anergy. We further our understanding of the immunopathology of sarcoidosis and point to novel therapeutic targets.

## Introduction

Sarcoidosis is a systemic inflammatory disease characterized by non-caseating granulomas. Granuloma formation and maintenance involves dynamic interaction among both adaptive and innate immune cells likely influenced by genetic risk, environmental stimuli and persistent foreign or self-antigens (1). Sarcoidosis susceptibility and etiology are both poorly understood and likely vary by ethnic background and environmental exposure (2–6). In fact, sarcoidosis is often termed an “immune paradox”, as both inflammation at disease sites and peripheral anergy to recall antigens are observed (7). Anergy, a mechanism of tolerance to suppress self-reactive lymphocytes, is consistent with sarcoidosis responding to standard autoimmune disease treatments and sharing both clinical presentation (1) and a genetic and molecular risk profile with several autoimmune disorders (8, 9). However, T cell reactivity to multiple mycobacterial proteins (10, 11) and, in some cases, response to antimycobacterial therapy (CLEAR trial) (12) also suggest infectious etiology.

Whereas past studies suggested compartmentalization of inflammation within diseased organs, recent findings suggest gene expression in granulomas is reflected in circulating immune cells (13–15). Two small RNA sequencing studies, one of monocytes (14) and one of regulatory T cells (16) have been published, as have other gene expression studies of tissue or blood using PCR- or microarray-based technologies. These studies of heterogeneous cell mixtures dilute cell-specific transcriptional signatures and thus, cell-type specific effects are not detectable.

It was thus our goal to characterize immune-cell-specific pathways that are dysregulated in the periphery of sarcoidosis patients *via* cutting-edge technology and analytics in the only single-cell RNA-sequencing (scRNA-seq) study and one of the largest transcriptomic studies in sarcoidosis to-date. We interrogated the transcriptomes of single cells and thus assessed hundreds of observations per sample for multiple immune cell subtypes. In doing so, we aimed not only to verify that both innate and adaptive signatures are present in the blood, but also to offer insight into points of immune dysfunction.

## Materials and Methods

### Cohort Characteristics

Participants include 35 consecutively evaluated sarcoidosis cases and 13 healthy controls recruited *via* community outreach and evaluated at the Sarcoidosis Research Unit of the Oklahoma Medical Research Foundation. The unit does not provide direct clinical care or diagnostic services to sarcoidosis patients. Therefore, our clinical staff, in consultation with our advisors at the University of Oklahoma Health Sciences Center and Vanderbilt University, designed and standardized a protocol of medical record review to document the diagnosis and clinical features of sarcoidosis adhering to the World Association of Sarcoidosis and other Granulomatous Disorders (WASOG) and A Case Controlled Etiologic Study of Sarcoidosis (ACCESS) guidelines (17–21). Briefly, assessment consisted of a one-time visit, where, after providing informed consent, subjects donated a blood sample (78.5 ml) and completed surveys of demographics, medication usage, and clinical histories. Patients provided authorization to obtain their medical records, which were reviewed to confirm biopsy and chest imaging reports compatible with a diagnosis of sarcoidosis and the exclusion of other granulomatous diseases (17, 20, 22, 23). Medical records were used to verify the use of immunosuppressant medications at the time of visit, in particular steroids or disease-modifying antirheumatic drugs (adalimumab, azathioprine, golimumab, hydroxychloroquine, infliximab, methotrexate, or prednisone). A summary of clinical and demographic information can be found in **Table 1**.

**Table 1:** Demographic and clinical information of sarcoidosis patients and healthy controls.

	CASES	CONTROLS
**Demographic features**		
Gender n(%)		
Male	12 (34.3)	3 (23.1)
Female	23 (65.7)	10 (76.9)
Race n(%)		
European American	27 (77.1)	12 (92.3)
African American	6 (17.1)	1 (7.7)
Native American	2 (5.7)	0 (0)
Mean age in years (SD)	57 (10.8)	45 (12)
**Disease Activity n(%)**		
Active*	12 (34.3)	n/a
Not-active	23 (65.7)	n/a
**Disease Duration n(%)**		
Chronic† (>2 years)	15 (42.8)	n/a
Persistent (>5 years)	11 (31.4)	n/a
**Organ Involvement n(%)§,‡**		
Lungs	35 (100)	n/a
Extra-thoracic lymph nodes	14 (40)	n/a
Calcium/Vitamin D	7 (20)	n/a
Liver	4 (11.4)	n/a
Spleen	4 (11.4)	n/a
Eyes	4 (11.4)	n/a
Skin	3 (8.6)	n/a
Bone/Joints	3 (8.6)	n/a
Bone marrow	2 (5.7)	n/a
Kidney	2 (5.7)	n/a
ENT	1 (2.9)	n/a
Nervous system	1 (2.9)	n/a
Heart	1 (2.9)	n/a
Other	1 (2.9)	n/a
**Medication usage at collection n(%)§,ll**		
Prednisone	13 (37.1)	1 (7.7)
Methotrexate	4 (11.4)	n/a
Hydroxychloroquine	3 (8.6)	n/a
Azathioprine	2 (5.7)	n/a
Infliximab	2 (5.7)	n/a
Adalimumab	1 (2.9)	n/a
Golimumab	1 (2.9)	n/a

*Defined as having exhibited (per medical records) novel organ involvement or a decrease in forced vital capacity of >10% in the last 12 months. ^†^Defined as unresolved disease lasting longer than 2 years. ^‡^According to recommendations (18, 19). ^§^A subject may have multiple organs affected or take multiple medications. ll Medications may have been taken for any cause, not exclusively for treatment of sarcoidosis.

### Single-Cell RNA-Sequencing

Cell capture was performed using the 10x Genomics Chromium system, a novel technology that utilizes high-throughput microfluidics to automatically encapsulate individual cells within oil droplets, each droplet containing a uniquely labeled DNA barcode bead. Single-cell 3’ transcriptomes originating from each droplet were recovered following sequencing on an Illumina HiSeq 3000, according to the 10x Genomics Chromium protocol. Briefly, peripheral blood mononuclear cells (PBMC) were collected using LSM (Gibco). No further sorting of PBMC was performed after collection. Fresh PBMC (target 4,000/patient) were loaded at a concentration of 1000 cells/µl into a 10x Genomics Single Cell A v2 chip into the Chromium controller. Following single cell emulsion generation, uniquely identifiable first strand template single-cell cDNA libraries were individually generated from each oil-encapsulated cell by emulsion PCR. After emulsion was broken, the second cDNA strand was generated and Illumina compatible adapters were ligated. Following qPCR quantification, final libraries were loaded onto single respective lanes of a HiSeq 3000 using read lengths of 26 bp for the first read, 98 bp for the second read, and an 8 base index read. Upon completion of sequencing the raw bcl files were processed using the 10x Genomics Cell Ranger (v3.0.2) informatics pipeline.

We obtained single-cell transcriptomes from 98,741 cells. After filtering of platelets and erythrocytes as well as additional quality control methods, 53,756 cells were retained for further analyses. Using a standard analysis pipeline [Seurat (24)], cells were clustered by similar gene expression and differential expression (DE) analysis was performed on each cell type independently. Gene expression was normalized within each cell cluster regardless of sample source. A subset of subjects did not contribute cells following quality control, resulting in a final sample of 33 cases (47,276 cells) and nine controls (6,480 cells) for DE analyses. For further technical details, see **Supplemental Methods**. We identified 3759 unique DE genes (**Figure S2**; **Table S2**), a large portion of which were cell-type specific (1578/3759, 42.0%).

To characterize cell-specific sarcoidosis-associated inflammatory pathways, we utilized a commercial software package, Ingenuity Pathway Analysis (IPA) to intersect DE genes with known biological functions. In addition, we utilized causal analysis approaches with IPA to identify upstream regulators experimentally determined to affect gene. For further technical details, see **Supplemental Methods**.

## Results

### Characterization of Demographic, Clinical, and Transcriptomic Data

Our cohort was predominantly European American (**Table 1**). Composition of patient and control groups did not differ by race or gender, but patients were older than controls on average (p=0.003) and thus age adjustment was included as part of scRNA-seq analyses. Sarcoidosis cases were 10.6 years post biopsy-proven diagnosis on average and had a wide range of organs affected, most frequently the lungs and extra-thoracic lymph nodes. Roughly half had unresolved disease lasting longer than 2 years and about a third had active disease at the time of their visit. A large proportion of patients were on immunosuppressive treatment at the time of sample collection. The proportion of subjects with active and/or chronic disease receiving immunosuppressants is similar to other reports (25, 26). To verify previous reports that treatment effects did not significantly alter results (13), we compared enriched pathways in treated and untreated patients (reported below).

**Figure 1:**
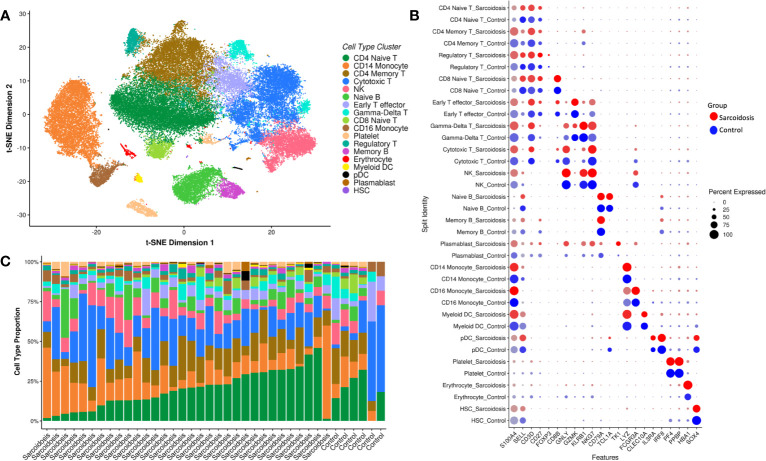
Eighteen cell types identified from PBMCs. Single-cell RNA-seq data is visualized by t-SNE plot of 53,756 human PBMCs colored by predicted cell type **(A)**. **(B)** Expression of marker genes of cell type by both average normalized expression (intensity of color) and percent of cells in that type expressing the marker gene (size of circle) in both sarcoidosis patients (red) and controls (blue). Bar charts **(C)** display the relative abundances of cells per type in patients and healthy controls that contributed at least 20 cells. While cell composition varies across individual, most subjects show large populations of CD4 T cells (dark green and brown), cytotoxic T cells (medium blue), CD14 monocytes (orange), and NK cells (pink).

To characterize cell-type-specific transcriptomes, we generated scRNA-seq profiles of over 100,000 PBMC. Following quality control measures, we identified 18 cell clusters (**Figure 1A**, **Supplemental Methods**), all of which could be assigned cell identities by canonical marker genes (**Figure 1B**, **S1**, **Supplemental Methods**). Thirteen cell types contained at least 1,000 cells (**Table S1**); others, including plasmacytoid dendritic cells (pDC, n=142) and plasmablasts (n=137) had only moderate representation in our samples (**Table S1**) and were thus excluded from downstream analyses. Each subject contributed an average of 1,280 cells that could be identified, although composition varied by individual (**Figure 1C**).

### Monocyte Activation and Trafficking Is Limited to Classical Monocytes

**Figure 2:**
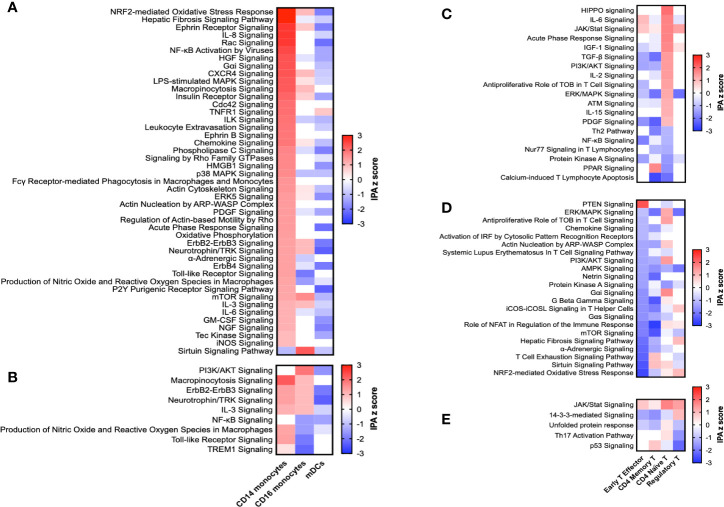
Select enriched pathways in monocyte and T cell subsets. Pathways significantly enriched by IPA are presented for CD14 monocytes **(A)**, CD16 monocytes **(B)**, CD4 naïve T cells **(C)**, Early T effector cells **(D)**, and regulatory T cells **(E)**. For convenience, IPA z scores of those pathways in similar cells are included in each graph. A pathway was defined as enriched if its IPA z score was above 1 or below -1, it had a significant p value (<0.05), and at least 5% of the genes in the pathway were also DE.

**Figure 3:**
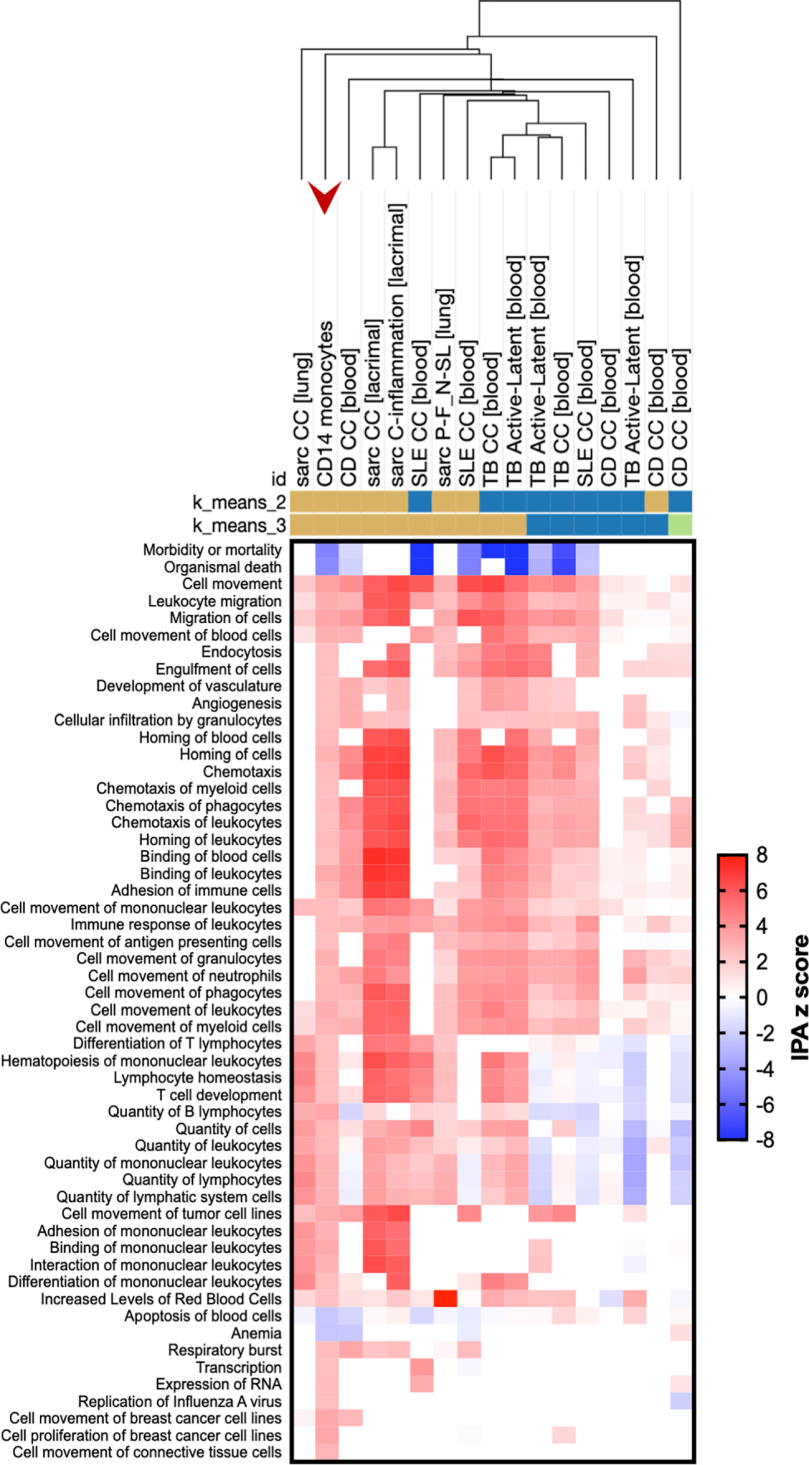
Sarcoidosis CD14 monocytes demonstrate enriched pathways similar to both infectious and autoimmune disease. Genes differentially expressed in sarcoidosis CD14 monocytes (red arrow) compared to those from controls were compared to publicly available experimental data from human diseases *via* the Analysis Match function of IPA. Included datasets are sarcoidosis (sarc), tuberculosis (TB), Crohn’s disease (CD), or systemic lupus erythematosus (SLE) data sets with the highest overall z score of similarity. Data sets were also limited to those from the HumanDisease data bank of either case-control or disease severity comparisons. Hierarchical clustering was performed utilizing the Morpheus tool (Broad Institute; https://software.broadinstitute.org/morpheus/) with the following parameters: one minus Pearson correlation, single linkage method, clustering of columns. K-means clustering was performed 20 times for each number of clustering and representatives of most common clusters are shown. Clustering was performed on all data; here, rows are limited to diseases and functions. From left to right, the following data sets are included: Case-control analysis (CC) of lung tissue in sarcoidosis, GSE16538.GPL570.test1; CC analysis of CD14 monocytes from this study of sarcoidosis, GSE132338; CC of peripheral blood in CD, GSE3365.GPL96.test1; CC of lacrimal gland in sarcoidosis, GSE105149.GPL570.test3; CC of lacrimal gland in sarcoidosis, GSE105149.GPL570.test7; CC of peripheral blood in SLE, GSE22098.GPL6947.test1; analysis of lung tissue from progressive, fibrotic (P-F) sarcoidosis vs. nodular, self-limiting (N-SL) sarcoidosis, GSE19976.GPL6244.test1; CC of peripheral blood in SLE, GSE50635.GPL6244.test2; CC of peripheral blood in TB, GSE19439.GPL6947.test2; analysis of peripheral blood of active vs. latent TB cases, GSE19439.GPL6947.test3; analysis of peripheral blood of active vs. latent TB cases, GSE19444.GPL6947.test3; CC of peripheral blood in TB, GSE19444.GPL6947.test2; CC of peripheral blood in SLE, GSE49454.GPL10558.test1; CC of peripheral blood in CD, GSE83381.GPL11154.DESeq2.test4; analysis of peripheral blood of active vs. latent TB cases, GSE28623.GPL4133.test3; CC of monocytes in CDGSE86434.GPL10558.test7; CC of peripheral blood in CD, GSE86434.GPL10558.test10.

Both animal models and patient studies have confirmed the importance of monocytes, the circulating precursors of macrophages, in the etiology and pathology of sarcoidosis (27). We, like others (14), found altered expression of genes important in activation (pattern recognition receptors and signaling molecules) and migration (adhesion molecules and chemokine receptors) in monocytes isolated from sarcoidosis patients compared to those from controls. We identified two distinct monocyte populations, classical (CD14) and non-classical (CD16) monocytes, both of which are considered pro-inflammatory, but differ in carrying out phagocytosis and activating T cells (28). Specifically, we confirmed previously reported enhancement of toll-like receptor (TLR) signaling (**Figure 2A**) and DE of TLR-signaling associated genes *TLR2*, *TNFAIP3*, and *NFKBIZ* as well as other pattern recognition receptors *CD163*, *FCN1*, *CLEC4A*, and *CLEC12A* in CD14 monocytes (**Table S2**). Signaling pathways classically known to affect monocytes activation were enriched in these cells as well, including IL-8, Rac/Cdc42, LPS-stimulated and p38 MAPK, TNFR1, chemokine, GM-CSF, and IL-6 signaling (**Figure 2A**). Additionally, pathways more recently found to play a role in monocyte activation, such as HMGB1 (29) and ephrin receptor (30) signaling, were also upregulated along with IL-3 signaling, previously associated with models of sepsis and two prototypical autoimmune diseases, systemic lupus erythematosus (SLE) and multiple sclerosis (31). Activated classical monocytes have been shown to undergo cytoskeletal rearrangements *via* RhoGTPases for extravasation (28); we saw upregulation of actin- and Rho-based motility pathways along with leukocyte extravasation signaling in these cells. Supporting the finding of increased trafficking, adhesion molecule *PECAM1* and integrin subunits *ITGB1* and *ITGAM* (CD11b), a gene known to play a role in SLE (32), were all upregulated. Downstream pathways were also enriched; CD14 monocytes upregulated acute phase response, iNOS, and mTOR pathways. We compared our findings to other datasets within the literature and found similarities to other sarcoidosis data sets as well as a prototypical infectious granulomatous disease, tuberculosis, a granulomatous autoimmune disease, Crohn’s, and SLE (**Figure 3**). Our findings show that classical monocytes in sarcoidosis, similar to other diseases, experience persistent innate activation, migration, and differentiation to antigen-presenting cells.

We found multiple lines of evidence that persistent CD14 monocyte activation may be enhanced by lack of regulatory mechanisms. HIF-1*α* expression has recently been found to be upregulated in sarcoidosis CD14 monocytes and associated with regulation of IL-1β and IL-17 production (33); here, we confirmed upregulation of *HIF1A* in these cells. ERK5 signaling, shown to be associated with monocyte inflammation and pro-inflammatory cytokine production in models of infection and ischemia (34), was also upregulated in CD14 monocytes. Finally, we saw upregulation of mTOR in CD14 monocytes; induced overactivity of this pathway was sufficient to generate spontaneous granulomas in a recent mouse model (35).

To further investigate the cause of dysregulation in these pathways, we utilized publicly available experimental data (DiseaseLand libraries, OmicsSoft, qiagenbioinformatics.com/diseaseland) and predictive modeling of upstream regulators and found evidence of master regulators of inflammation, fibrosis, and autophagy. In this proprietary algorithm (IPA), the effects of known regulators are curated from literature and compared to gene expression changes. For example, here, among other genes, *STAT1*, *IL10RA*, and *PTEN* were underexpressed in sarcoidosis CD14 monocytes compared to controls while *CXCR4*, *ATG12*, *HIF1A*, *CCR1*, and *IER3* were overexpressed, consistent with the effects of TGFB1 signaling. Utilizing this method predicted 39 potential upstream regulators activated or inhibited in sarcoidosis CD14 monocytes with confidence; lipopolysaccharide, TGFB1, STAT3, and RICTOR, a component of the mTOR complex, were among the top 10 (**Figure S3**; **Table S3**). TGFβ is widely recognized as a core pathway of fibrosis, and mTOR signaling is a central regulator of autophagy. Treatment was not responsible sarcoidosis-associated CD14 monocyte dysregulation; treatment led to the reversal of the direction of the enrichment of the majority of pathways (**Figure S4**). These results suggest that sarcoidosis monocytes may receive no more innate stimuli than those of healthy controls, but aberrant regulatory pathways associated with fibrosis and autophagy induce persistent hyperactivation. Current immunosuppressive therapies may be somewhat effective in controlling this hyperactivation, but more investigation of larger cohorts is necessary.

While previous studies have suggested a role of CD16 monocytes (36), we found little evidence of cell-type-specific differences between these cells in sarcoidosis cases and healthy controls. Like CD14 monocytes, pathways known to affect monocyte activation were upregulated in CD16 monocytes, including PI3K/AKT signaling (**Figure 2B**). However, other inflammatory pathways, including NF*κ*B, TLR, and TREM1 signaling were downregulated. IL-3, neurotrophin/TRK (37), and macropinocytosis signaling were upregulated in both monocyte types, while shared pathways of production of nitric oxide and reactive oxygen species and TLR signaling were downregulated in CD16 monocytes. In contrast to classical monocytes, CD16 monocytes demonstrated predicted inhibition of a single upstream regulator, CSF3 (G-CSF; **Table S3**). These results suggest future studies should be performed on isolated monocyte subsets to localize inflammatory signals.

### CD4 Naïve T Cells Display Markers of Non-TCR-Mediated Activation, Apoptosis, and Differentiation Dysregulation

Sarcoidosis models and patient studies also point to T cells as key players in the pathogenesis of sarcoidosis, indicating an interplay between adaptive and innate immunity (9, 27). We found evidence of CD4 naïve T cell activation in sarcoidosis as well as enrichment of regulatory pathways, suggesting a persistent inflammatory response of potentially infectious or autoimmune origin. Specifically, CD4 naïve T cells displayed upregulation of JAK/STAT, PI3K/AKT, and ERK/MAPK signaling, suggesting general activation (**Figure 2C**). As expected for naïve cells, we found no evidence indicative of antigen-driven T cell stimulation (**Figure 2C**, **Table S2**). Indeed, only antigen-independent stimulatory pathways capable of affecting naïve CD4 T cells prior to antigen recognition, such as IL-2, IL-15, IL-6, and PDGF signaling, were enriched in these cells.

As in CD14 monocytes, we also found evidence of dysregulation of multiple regulatory mechanisms. First, multiple pathways indicated a loss of mechanisms of apoptosis in CD4 naive T cells. Signaling *via TOB1*, a negative regulator of T cell proliferation and cytokine transcription, was upregulated. ATM signaling, known to maintain naïve T cell survival, was upregulated, and T lymphocyte apoptosis was downregulated. PPAR signaling, which may regulate both T cell survival and differentiation (38), was also downregulated. Other regulators of T cell differentiation, including PDGF (39) and IGF-1 (40) signaling, known to regulate T_reg_ proliferation and function in, among others, autoimmune diseases, were enriched. Similarly, both HIPPO signaling and TGFβ signaling, known to affect Th17/T_reg_ differentiation (41), were enriched. Together, these results suggest dysregulation of both apoptosis and differentiation mechanisms consistent with other autoimmune diseases that may inhibit resolution of the initial immune response in sarcoidosis. Treatment reversed the direction of most of these enriched pathways, suggesting an inhibitory role (**Figure S4B**). Interestingly, treatment also upregulated Th2 signaling in these cells, potentially promoting differentiation to this effector T cell subset.

### Effector T Cells Upregulate Anergy-Associated Genes and Suppress T Cell Receptor Signaling

T cell anergy is a mechanism of peripheral tolerance that is classically defined as a hyporesponsive state established in T cells when antigen is sensed in the absence of co-stimulation such as CD28 binding. Anergy prevents cell proliferation and cytokine production in response to subsequent antigen encounter *via* blockade of T cell receptor (TCR) signaling and is thought to be protective against autoimmunity. In sarcoidosis, anergy is observed specifically by lack of reaction to skin antigen tests and peripheral blood exposure *ex vivo* to recall antigens (42). Mechanisms of this observed anergy are poorly understood, but both compartmentalization of immune competent cells to affected tissues and T_reg_ or effector T cell dysfunction have been proposed. Here, we find enrichment of pathways classically associated with anergy (43). Specifically, early T effector cells (ETE) from sarcoidosis patients had significant downregulation of TCR signaling (labeled “SLE T cell signaling pathway” here) and ICOS-ICOSL signaling, as well as PI3K/AKT, ERK/MAPK, NFAT, ERBB2 (TOB), sirtuin, and mTOR signaling (**Figure 2D**), relative to controls.

Two different systems are widely utilized to induce anergy in T cells: treatment with ionomycin (44) or stimulation with anti-TCR (45)/anti-CD3 (46). To further validate an anergy phenotype in our ETE, we compared our findings to three microarray studies utilizing these methods. Of the 392 anergy-associated genes, only 18 (4.6%) were shared by at least two studies, suggesting a lack of consensus of an anergy “signature”. However, a large portion of anergy-associated genes (115; 29.3%) were DE in sarcoidosis ETE (**Table S4**).

When we assessed DE genes for potential gene, protein, or mRNA upstream regulators (IPA, digitalinsights.qiagen.com), CD3 and CD40LG were the two most significantly associated, inhibited, transcriptional regulators. Similarly, causal network analysis found the TCR-CD3 complex the most likely master regulator of all ETE DE genes. As our cells were untouched by antibodies, we can conclude our findings were not due to effects of labeling. Differences in proportion of ETE cells between cases and controls or subsets of cases were not observed. Enriched pathways were not driven by treatment, as treatment subset analyses yielded almost no enriched pathways (data not shown). This evidence suggests anergy in sarcoidosis is driven by persistent inhibition of TCR-mediated T cell activation in peripheral ETE *via* enrichment of anergy-related pathways, potentially as a mechanism to prevent activation of self-reactive T cells in the presence of persistent cognate self-antigen.

### Sarcoidosis T Cell Dysregulation Is Potentially Aggravated by Changes in Regulatory T Cell Survival and Differentiation

Regulatory T cells (T_reg_) suppress T cell subsets that have been activated by either infectious or autoimmune antigens through multiple mechanisms, including depletion of local IL-2 through IL2Rα, secretion of granzymes to induce cytolysis, and CTLA-4-dependent suppression of antigen-presenting cells. T_reg_ from sarcoidosis patients have been shown to have reduced suppressive capacity *ex vivo* (47), increased expression of *FOXP3* (16), decreased *CTLA4* (48), and altered expression of genes in the p53 pathway (16). We found downregulation of the p53 pathway in these cells (**Figure 2E**) along with DE of p53 pathway genes *ATM*, *BAX*, *BCL2*, *PIAS1*, and *PRKDC* (**Table S2**). Both previously-found and novel cell death genes were enriched in T_reg_ (e.g., *BCL2*, *CASP1*; **Table S2**), substantiating the observation that sarcoidosis T_reg_ have decreased survival (49). Signaling *via* TNFR2, a known regulator of T_reg_ function (50) did not meet criteria for enrichment *via* IPA analyses; however, members of the TNFR2 signaling pathway were differentially expressed in T_reg_, including *TANK* and *TNFAIP3*. We observed enrichment of Th17 signaling, suggesting dysregulation of the balance of Th17/T_reg_ differentiation. Finally, we confirmed our findings were not driven by treatment, as treated patients did not display any enriched pathways when compared to untreated patients (data not shown).

## Discussion

**Figure 4:**
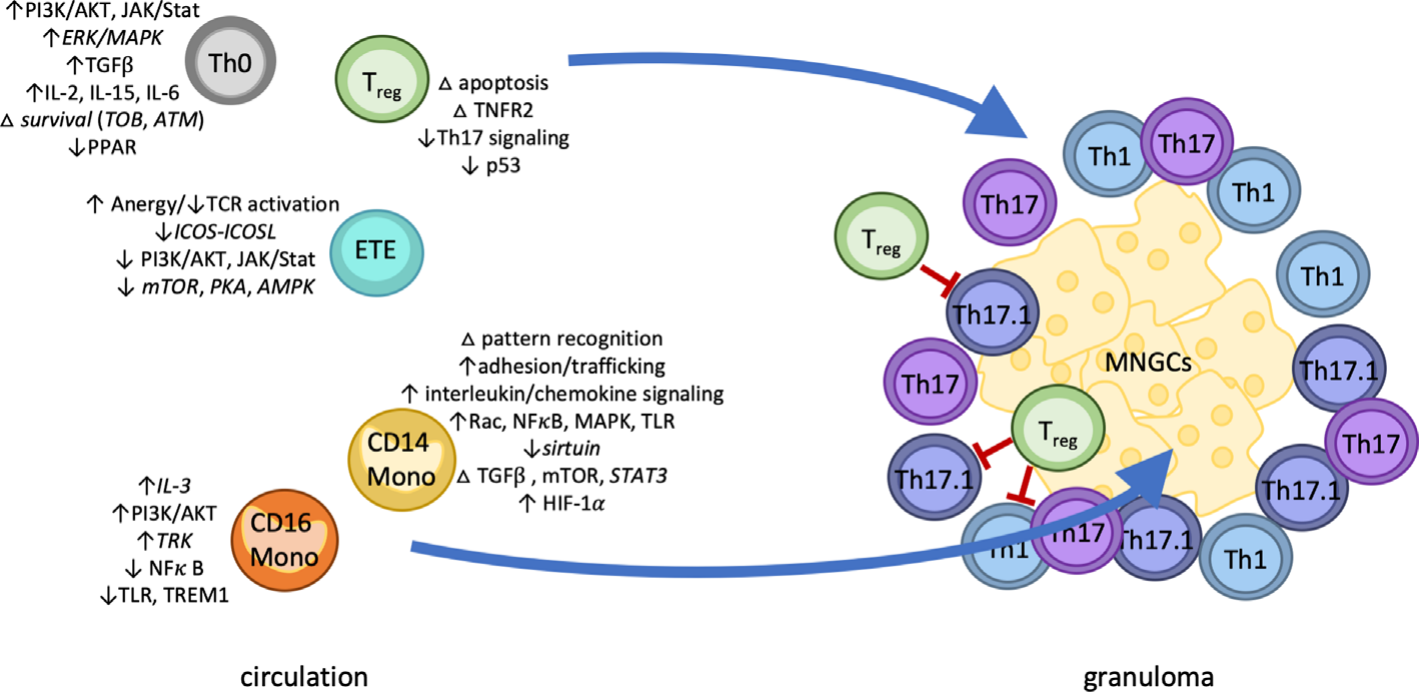
The four pillars of sarcoidosis model. Classical monocytes upregulated distinct markers of activation including adhesion molecules, pattern recognition receptors, and chemokine receptors, as well as enrichment of immunoregulatory pathways HMGB1, mTOR, and ephrin receptor signaling. Predictive modeling implicated TGFβ and mTOR signaling as drivers of persistent classical monocyte activation. In contrast, CD16 monocytes displayed both up- and down-regulation of a small number of inflammatory pathways. Sarcoidosis T cells subsets also displayed patterns of dysregulation. CD4 naïve T cells were enriched for markers of apoptosis and Th17/T_reg_ differentiation, while effector T cells showed enrichment of anergy-related pathways. Differentially expressed genes in regulatory T cells suggested dysfunctional p53, cell death, and TNFR2 signaling. We therefore hypothesize sarcoidosis pathology is marked by four distinct cell-specific effects: 1) persistent hyperactivation of innate immunity *via* classical monocytes 2) in combination with CD4 naïve T cell activation and 3) exacerbated by regulatory T cell dysfunction and potentially 4) self-reactive effector T cells potentially suppressed by anergy. Abbreviations: Th0, CD4 naïve T cell; ETE, early T effector; T_reg_, regulatory T cells; MNGCs, multi-nucleated giant cells; Th1/17/17.1, differentiated helper T cells; LN, lymph node; CD14 mono, classical (CD14+) monocytes; CD16 mono, non-classical (CD16+) monocytes. Italics indicate novel discovery of basal gene- or protein-level enrichment of the indicated pathway in the indicated cell type in sarcoidosis.

Sarcoidosis is an enigmatic disease involving established genetic predisposition and immune dysregulation but an otherwise unknown etiology. Here, in one of the largest gene expression studies of sarcoidosis, we apply cutting-edge technology for examining the transcriptomic profiles of single cells in as natural and unmodified state as possible. Our objective was to pinpoint the differential gene expression between patients and controls to specific cell subtypes, identifying relevant dysregulation in specific peripheral immune cells. Our results substantiate immune dysregulation inherent to sarcoidosis that involves peripheral hyperactivation of both T cells and classical monocytes with subsequent migration into affected tissue. We solidify a number of commonalities between sarcoidosis and both infectious and autoimmune diseases, including persistent hyperactivation of innate immunity *via* classical monocytes as well as CD4 naïve T cell activation, regulatory T cell dysfunction, and enrichment of fibrosis-, autophagy-, and anergy-associated genes and pathways (51). We show evidence of novel dysregulation of both T cell and monocyte subsets and identify potential upstream regulators and regulatory mechanisms (summarized in **Figure 4**). Finally, our highly detailed, single-cell expression patterns provide multiple candidate pathways for targeted, bench-to-bedside treatments aimed at reestablishing normal functions of the main dysregulated cell types that we identified in sarcoidosis. Ephrin receptor signaling, upregulated in sarcoidosis CD14 monocytes, has been an attractive therapeutic target for both infection and cancer (52) and has recently been investigated as a therapy for Inflammatory Bowel Disease (53). A recent study found chloroquine, a drug commonly used in the treatment of autoimmune disease, could reduce HIF-1*α* levels in sarcoidosis alveolar macrophages and reduce IL-17 and IL-1β production in sarcoidosis PBMC in response to anti-CD3 (33). More investigation is needed to ensure chloroquine would effectively reduce the enrichment of *HIF1A* seen here in classical monocytes. Additionally, blocking of activation of sarcoidosis classical monocytes by targeting GM-CSF with otilimab (GSK3196165) or IL-3 signaling with XMD8-92 (56) are suggested by our findings. Similarly, our findings in sarcoidosis CD4 naïve T cells suggest they may be effectively targeted by nintedanib, a multi-tyrosine kinase inhibitor approved for use in idiopathic pulmonary fibrosis that targets the JAK/STAT pathway and reduces the effects of TGFβ signaling (54). While sarcoidosis T_reg_ were not highly dysregulated here, the p53 pathway is the target of existing pharmacological therapies and should be further explored as a target of immunomodulation of sarcoidosis T_reg_. Additional avenues of investigation suggested by our findings include antagonists of HMGB1 (55), Tec kinases, PPAR, JAK/STAT, TGFβ, and mTOR inhibitors such as rapamycin (51), among many others.

While our study presents many novel insights, we acknowledge they are not without certain limitations. Our patient population is more representative of an outpatient than a hospital-based or a research sample with targeted enrollment by phenotype, and thus comprises a less homogeneous patient population, suggesting our results are applicable across multiple disease subtypes, even in the presence of treatment. We also acknowledge that there has been some evidence of distinct immune dysregulation in circulating and tissue-resident immune cells in sarcoidosis. However, as we and others have shown (13–15), the immune dysregulation characteristic of sarcoidosis is present in circulating immune cells, collection of which is a much less invasive and cost-effective option for new diagnostic tools. The collection and analysis of scRNA-seq data is still a developing field; however, multiple studies have now been published with the standard analytical techniques we utilized. Finally, as with all such studies, there is concern about the influence of any number of factors on the levels of gene expression. One potential source of altered expression is stimulation or manipulation of the cells themselves; thus, we chose to use the 10x technology instead of flow cytometry to isolate cells, minimizing contact and manipulation. The other major potential source of altered gene expression is medication use. Our assessment of treatment effects is consistent with the many gene expression studies done to date that show modest effects of treatment on specific genes but do not hinder the ability to find meaningful, biologically relevant differences. This has been shown to be particularly true in single cell studies as the number of observations per patient are increased by 100-fold, thus maximizing power while minimizing false positives.

To date, no other single-cell characterization of circulating immune cells in sarcoidosis has been published. In this study, we have shown, using scRNA-seq, that cell type-specific differences exist in subsets of PBMC of sarcoidosis cases versus healthy controls. Our findings confirm previous findings in both T cells and monocytes and offer novel insights into the source of persistent immune dysregulation. We offer strong evidence that sarcoidosis is a systemic disease and that relatively noninvasive access to circulating immune cells offers the potential for novel and powerful diagnostics. We also show novel evidence of the mechanisms by which immune dysregulation in sarcoidosis patients persists, regardless of the initial stimuli. This new mechanistic insight offers practical targets for novel and repositioned pharmaceutical intervention.

## Data Availability Statement

The datasets presented in this study can be found in online repositories. The names of the repository/repositories and accession number(s) can be found below: https://www.ncbi.nlm.nih.gov/geo/ GSE132338.

## Ethics Statement

The studies involving human participants were reviewed and approved by Institutional Review Board of the Oklahoma Medical Research Foundation. The patients/participants provided their written informed consent to participate in this study.

## Author Contributions

Concept and Design: LG, RP, CM. Acquisition, Analysis, or Interpretation: LG, RP, AR, CL, KS, SD, WD, CM. Drafting: LG, RP, AR, CM. Revising: LG, RP, AR, UD, HB, AL, SD, WD, CM. All authors contributed to the article and approved the submitted version.

## Funding

This work was supported by grants from the Foundation for Sarcoidosis research (Chicago, IL), and the National Institutes of Health (R01HL113326-05, P30 GM110766-01, U54GM104938-06, R01HL117074, K24HL127301).

## Conflict of Interest

The authors declare that the research was conducted in the absence of any commercial or financial relationships that could be construed as a potential conflict of interest.

## Supplementary Material

Supplementary Figure 1 Prominent genes defining t-Distributed Stochastic Neighbor Embedding (t-SNE) clusters. Cells were clustered into presumptive cell types based on scRNA-seq profiles. Cells are colored in successive panels by scaled gene expression values for classical marker genes (refer to **Supplemental Methods**) for each subtype: CD4+ T (CD3D+, SELL^hi^), CD8+ T (CD3D+, CD8A/B+), NK cells (GNLY+, NKG7+), B cells (CD79A+), Naïve B cells (TCL1A+), CD14+ monocytes (LYZ+, FCGR3A-), CD16+ monocytes (LYZ+, FCGR3A+), and T_reg_ (FOXP3+). Other genes that assist in distinguishing further subtypes are also included.

Supplementary Figure 2 Genes differentially expressed in patient compared to control cell types. Volcano plot of 3759 differentially expressed gene/cell type combinations in scRNA-seq profiles. X-axis indicates the log2 fold change in sarcoidosis patients relative to healthy controls, as estimated by Seurat. Points are colored by cell type. The top 30 most differentially expressed genes are labeled.

Supplementary Figure 3 Predicted activated upstream regulators of CD14 monocytes from sarcoidosis patients. Shown are the predicted CD14 monocyte upstream regulators TGFB1 **(A)**, STAT3 **(B)**, and lipopolysaccharide **(C)**, with downstream molecules. Intensity of color in orange and blue molecules indicate level of confidence in predicted activation (e.g., STAT1) or inhibition, respectively. Color of lines indicates predicted relationships: orange, activation; blue, inhibition; yellow, findings inconsistent with state of downstream molecule (e.g., SP1); gray, unknown direction of prediction. When measured and predicted levels disagree, molecules are surrounded by a glow (not seen here). All shown analyses were significant (IPA).

Supplementary Figure 4 Enriched pathways in CD14 monocytes and CD4 naïve T cells are affected by immunosuppressive treatment. Pathways significantly enriched by IPA in case-control analyses are presented for CD14 monocytes **(A)** and CD4 naïve T cells **(B)**. IPA Z scores are included from subset analyses in which cells of the same type from patients treated with immunosuppressive medications compared to untreated patients (Treated-Untreated). A pathway was defined as enriched if its case-control analysis IPA Z scores was above 1 or below -1, it had a significant p value (<0.05), and at least 5% of the genes in the pathway were also DE.
