# Supplementary figures and images for: Single Cell Transcriptomics Implicate Novel Monocyte and T Cell Immune Dysregulation in Sarcoidosis
Source: Front Immunol. 2020 Dec 8;11:567342. doi: 10.3389/fimmu.2020.567342 (PMC7753017; doi:10.3389/fimmu.2020.567342)

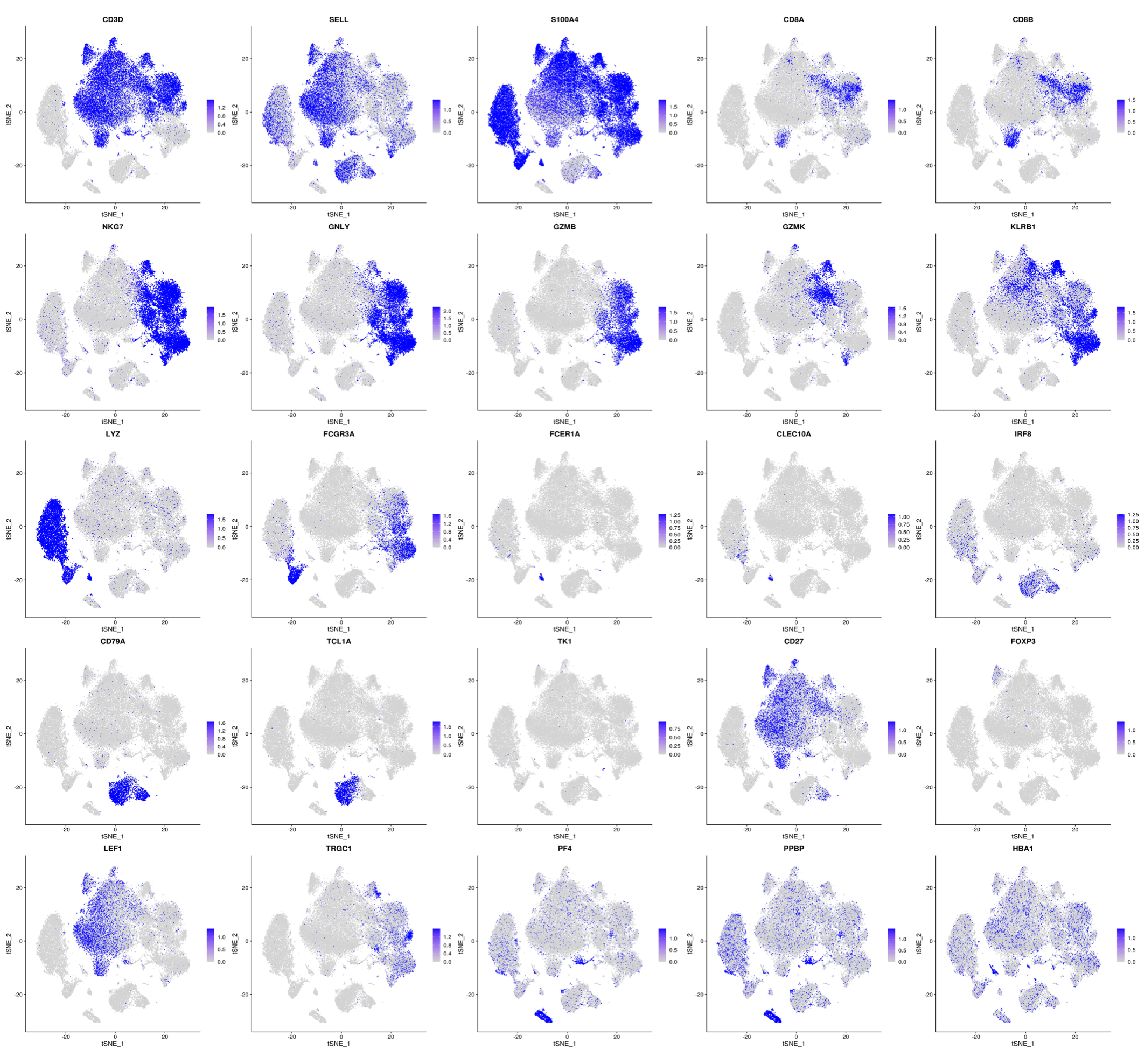

Supplement: Supplementary Figure 1 — Prominent genes defining t-Distributed Stochastic Neighbor Embedding (t-SNE) clusters. Cells were clustered into presumptive cell types based on scRNA-seq profiles. Cells are colored in successive panels by scaled gene expression values for classical marker genes (refer to Supplemental Methods ) for each subtype: CD4+ T (CD3D+, SELLhi), CD8+ T (CD3D+, CD8A/B+), NK cells (GNLY+, NKG7+), B cells (CD79A+), Naïve B cells (TCL1A+), CD14+ monocytes (LYZ+, FCGR3A-), CD16+ monocytes (LYZ+, FCGR3A+), and Treg (FOXP3+). Other genes that assist in distinguishing further subtypes are also included. [file DataSheet_2.zip › Supplementary Figure 1.tiff]

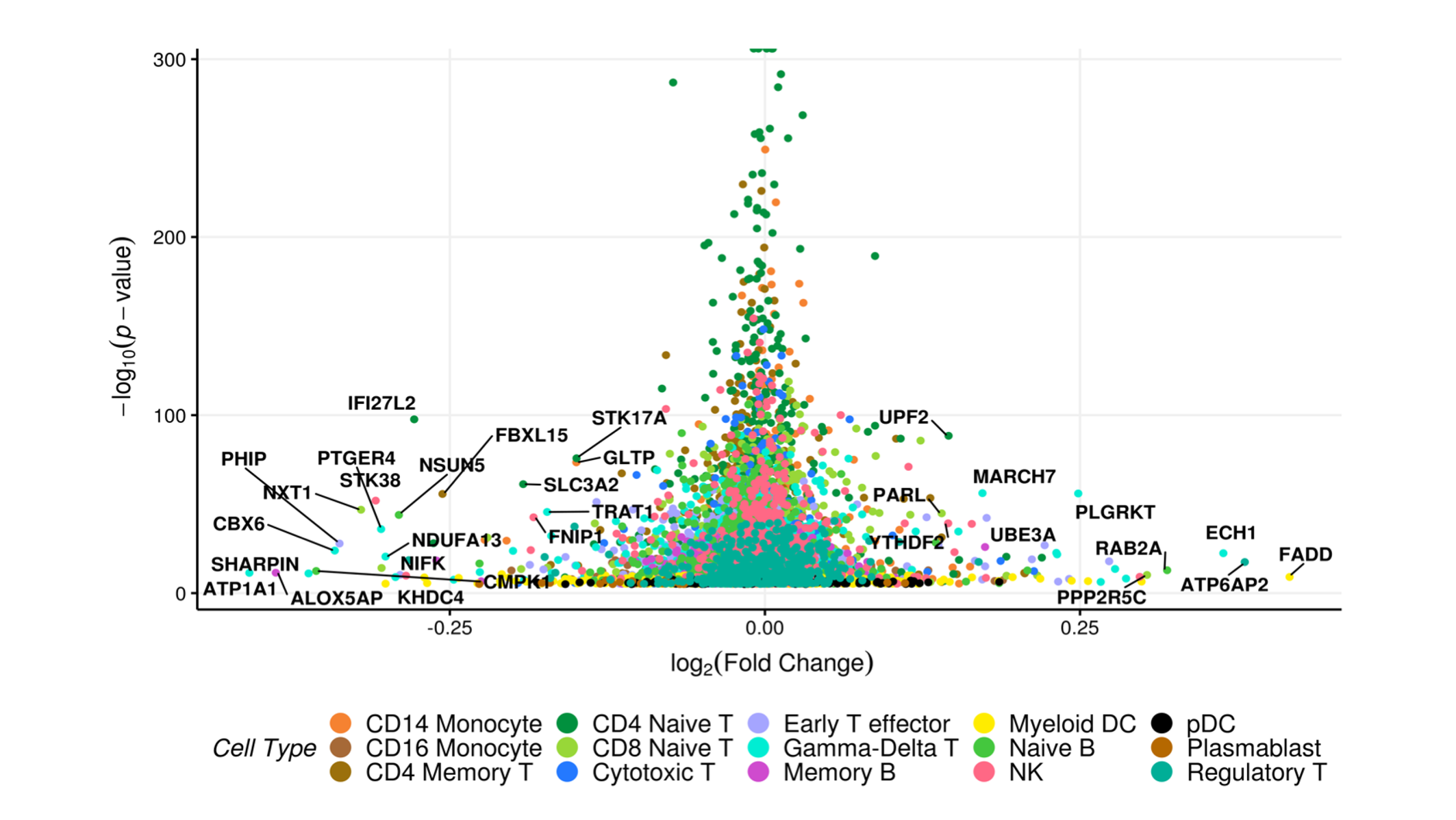

Supplement: Supplementary Figure 1 — Prominent genes defining t-Distributed Stochastic Neighbor Embedding (t-SNE) clusters. Cells were clustered into presumptive cell types based on scRNA-seq profiles. Cells are colored in successive panels by scaled gene expression values for classical marker genes (refer to Supplemental Methods ) for each subtype: CD4+ T (CD3D+, SELLhi), CD8+ T (CD3D+, CD8A/B+), NK cells (GNLY+, NKG7+), B cells (CD79A+), Naïve B cells (TCL1A+), CD14+ monocytes (LYZ+, FCGR3A-), CD16+ monocytes (LYZ+, FCGR3A+), and Treg (FOXP3+). Other genes that assist in distinguishing further subtypes are also included. [file DataSheet_2.zip › Supplementary Figure 2.tiff]

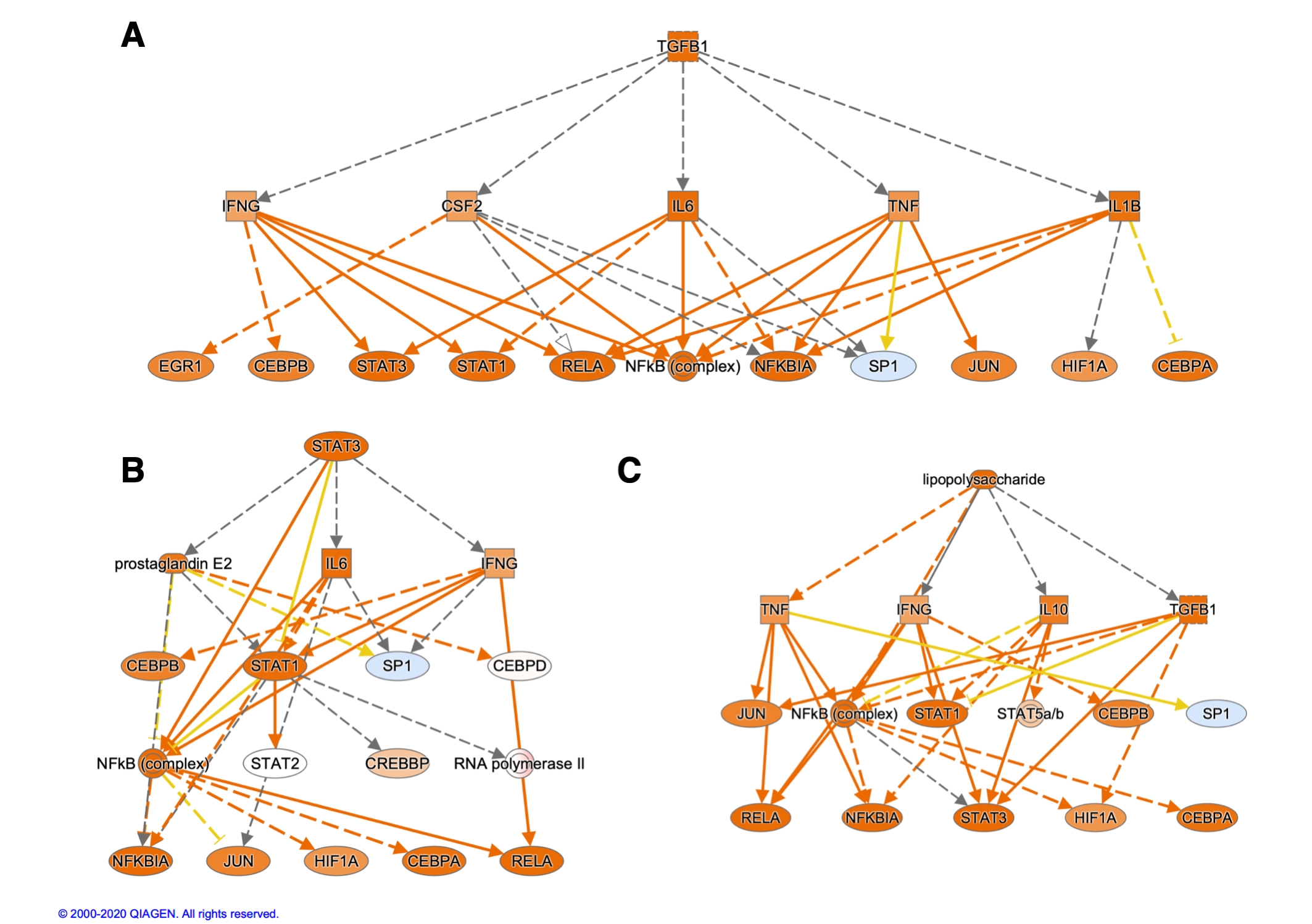

Supplement: Supplementary Figure 1 — Prominent genes defining t-Distributed Stochastic Neighbor Embedding (t-SNE) clusters. Cells were clustered into presumptive cell types based on scRNA-seq profiles. Cells are colored in successive panels by scaled gene expression values for classical marker genes (refer to Supplemental Methods ) for each subtype: CD4+ T (CD3D+, SELLhi), CD8+ T (CD3D+, CD8A/B+), NK cells (GNLY+, NKG7+), B cells (CD79A+), Naïve B cells (TCL1A+), CD14+ monocytes (LYZ+, FCGR3A-), CD16+ monocytes (LYZ+, FCGR3A+), and Treg (FOXP3+). Other genes that assist in distinguishing further subtypes are also included. [file DataSheet_2.zip › Supplementary Figure 3.tiff]

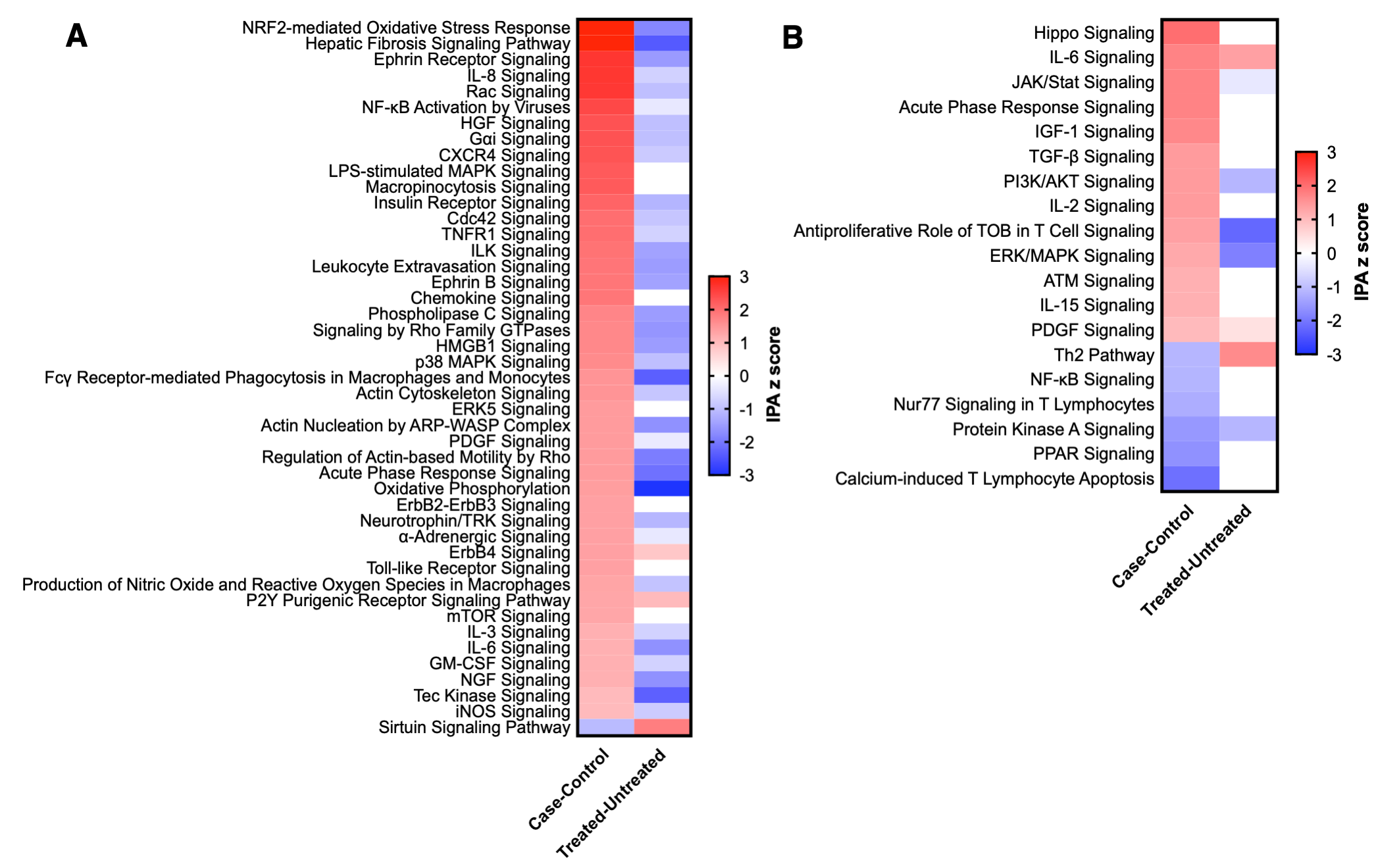

Supplement: Supplementary Figure 1 — Prominent genes defining t-Distributed Stochastic Neighbor Embedding (t-SNE) clusters. Cells were clustered into presumptive cell types based on scRNA-seq profiles. Cells are colored in successive panels by scaled gene expression values for classical marker genes (refer to Supplemental Methods ) for each subtype: CD4+ T (CD3D+, SELLhi), CD8+ T (CD3D+, CD8A/B+), NK cells (GNLY+, NKG7+), B cells (CD79A+), Naïve B cells (TCL1A+), CD14+ monocytes (LYZ+, FCGR3A-), CD16+ monocytes (LYZ+, FCGR3A+), and Treg (FOXP3+). Other genes that assist in distinguishing further subtypes are also included. [file DataSheet_2.zip › Supplementary Figure 4.tiff]
