# Supplementary material for: Single Cell Transcriptomics Implicate Novel Monocyte and T Cell Immune Dysregulation in Sarcoidosis
Source: Front Immunol. 2020 Dec 8;11:567342. doi: 10.3389/fimmu.2020.567342 (PMC7753017; doi:10.3389/fimmu.2020.567342)
Supplement: Supplementary file 3 [file DataSheet_3.docx]

Supplementary Material

# Supplementary Methods

1.1 Single-cell Differential Expression from PBMCs

Total cells sequenced (n=98,741) were first filtered for quality and technical artifacts; erythroid cells were also excluded. The Louvain algorithm identified nineteen major cell clusters by similar gene expression. Cell-type identities were assigned to these clusters on the basis of expression of cluster-specific marker genes. Differentially expressed (DE) genes between sets of individuals in the final set of cells (n= 53,756) were identified using functionality in R package Seurat v3.0(E1).

1.2 Single-cell RNA-seq quality control, cell type determination and differential expression analysis

We first removed individual cells if they met any of the following conditions: 1) Total number of genes with positive expression was below 200 (n=35,207) or above 2000 (n=413), 2) The proportion of mitochondrial reads was above 20% (n=1,217), or 3) the probability of being a cell doublet was estimated to be greater than 7.5% as estimated by the DoubletFinder method (E3) (n=3,284). Finally, 4849 cells originated from donors which did not meet our rigorous clinical definition of sarcoidosis. overlap of these sequencing QC filters permitted 53,756 total cells

To account for the potential of disease status to adversely bias cell type determination, we first performed comprehensive integration of separated control and sarcoidosis datasets using the Canonical Correlation Analysis (CCA)-based approach in Seurat(4). Briefly, control or sarcoidosis cells were log-normalized and the top 2000 variable features were identified. Integration anchors for both control and sarcoidosis cells were identified across these features using the *FindIntegrationAnchors* function, using 30 CCA dimensions. Finally, the data was integrated using the *IntegrateData* function, using the top 30 CCA dimensions. The resulting integrated data was the scaled, and the top 70 principal components were computed.

Clusters of cells were identified using the Louvain algorithm via the *FindNeighbors* and *FindClusters* functions, using the top 70 principal components and clustering resolution of 0.5. This resulted in the identification of 18 distinct clusters. For each cluster, we used the *FindMarkers* function in Seurat with default parameters to identify the top upregulated genes identifying each cluster. Cell types were determined using the expression of canonical cell-type-specific genes within these cluster-specific lists (Supplemental Methods Table 1). A total of 53,756 cells were confidently assigned to a cell type and were used for further analyses. The integrated data for these cells was then embedded via t-SNE (theta =0.2, perplexity=60) for visualization and confirmation of cell type expression patterns (Figure 1A).

| **Cell Type** | **Expression Pattern** | **References** |
| --- | --- | --- |
| CD4 Naïve T | CD3D+, CCR7+, SELL+, S100A4-,CD8A-,CD8B- | (E5) (E6) |
| CD4 Memory T | CD3D+, S100A4+, CCR7-, CD8A-, CD8B- | (E7) |
| Early T effector | CD3D+, GZMK+, GZMB- | (E8) |
| Regulatory T | CD3D+, CD27+, FOXP3+ | (E9) |
| Gamma-Delta T | CD3D+, TRGC1+ | (E10) |
| CD8 Naïve T | CD3D+, CD8A+, CD8B+, S100A4+ |  |
| Cytotoxic T | CD3D+, CD8A+, CD8B+, GZMB-, GZMK-, GNLY+ | (E11) |
| NK | CD3D-, NKG7+, GZMB+ | (E12) |
| CD14 Monocyte | LYZ+, FCGR3A- | (E13) |
| CD16 Monocyte | LYZ+, FCGR3A+ | (E14) |
| Myeloid DC | FCER1A+, CLEC10A+ | (E15), (E16) |
| pDC | GZMB+, IL3RA+ | (E17, E18) |
| Naïve B | CD79A+, TCL1A+ | (E19) |
| Memory B | CD79A+,TCL1A- | (E20) |
| Megakaryocyte | PF4+, PPBP+, CD79A+ | (E21) |
| Platelet | PPBP+, CD79A- |  |
| HSC | SOX4+ | (E22) |
| Erythrocyte | HBA1+ |  |

*Supplemental Methods Table 1. Cell-type determining markers.*

Tests of differential expression were accomplished by returning to the original, pre-integration transcriptome counts for cells restricted by cell type and subjects in the tested categories. Normalization and scaling was done with the *SCTransform* function (E23) in Seurat. During normalization, we regressed out coefficients corresponding to individuals’ age, sex, Cyclone cell cycle score (S – G2M), and total number of genes expressed per cell. This normalized, scaled data is used to calculate differential expression of genes between the cells defined by subtypes of contributing individuals using the Wilcoxon test built into the *FindMarkers* function in Seurat, using the following parameters: logfc.threshold=0.01.

1.3 Ingenuity Pathway Analyses

Ingenuity Pathway Analysis (IPA) is a commercial software package most commonly utilized to intersect DE genes with known biological functions and pathways maintain in the Ingenuity Knowledge Base, a collection of nearly 5 million experimental findings manually curated from either literature or third-party databases. Detailed information can be found at http://qiagen.force.com/KnowledgeBase/. Enriched pathways are identified via the Canonical Pathways function. The significance of the overlapping genes from DE genes and genes within the enriched pathway is reported as a p value, calculated by the right-tailed Fisher's Exact Test. In addition to a p value, Canonical Pathways reports the percent overlap (given as a ratio) and a z-score, a measure of activation or inhibition of a pathway based on the observed and predicted magnitude and direction of fold changes in overlapping genes. Calculations of z scores have been described in detail(E24). Here, a pathway was defined as enriched if its IPA Z score was above 1 or below -1, it had a significant p value (<0.05), and the overlap of DE and pathway genes was at least 5%.

Causal analytics algorithms also available in IPA have been described in detail(E24). We utilized three of these algorithms, Upstream Regulator Analysis, Causal Network Analysis, and Diseases and Functions. Briefly, Upstream Regulator Analysis determines likely upstream regulators, including transcription factors, genes, and small molecules, that are connected to dataset genes through a set of direct or indirect relationships. Causal Network Analysis further connects upstream regulators to dataset molecules but includes intermediate regulators; it is thought to generate a more complete list of master regulators, or possible causes of all observed expression changes. Molecules were defined as an upstream regulator or a master regulator if it had a significant p value and could be predicted to be activated or inhibited (activation z score of above 2 or below -2). Diseases & Functions uses causal analytics algorithms to infer impact on biological functions and diseases downstream of DE genes in a dataset.

# Supplementary References

E1. Butler A, Hoffman P, Smibert P, Papalexi E, Satija R. Integrating single-cell transcriptomic data across different conditions, technologies, and species. *Nat Biotechnol* 2018; 36: 411-420.

E2. Scialdone A, Natarajan KN, Saraiva LR, Proserpio V, Teichmann SA, Stegle O, Marioni JC, Buettner F. Computational assignment of cell-cycle stage from single-cell transcriptome data. *Methods* 2015; 85: 54-61.

E3. McGinnis CS, Murrow LM, Gartner ZJ. DoubletFinder: Doublet Detection in Single-Cell RNA Sequencing Data Using Artificial Nearest Neighbors. *Cell Syst* 8 (2019); 329-337 e4.

E4. Stuart T, Butler A, Hoffman P, Hafemeister C, Papalexi E, Mauck WM, 3rd, Hao Y, Stoeckius M, Smibert P, Satija R. Comprehensive Integration of Single-Cell Data. *Cell* 2019; 177: 1888-1902 e1821.

E5. Mullen KM, Gocke AR, Allie R, Ntranos A, Grishkan IV, Pardo C, Calabresi PA. Expression of CCR7 and CD45RA in CD4+ and CD8+ subsets in cerebrospinal fluid of 134 patients with inflammatory and non-inflammatory neurological diseases. *J Neuroimmunol* 2012; 249: 86-92.

E6. Elyahu Y, Hekselman I, Eizenberg-Magar I, Berner O, Strominger R, Schiller M, Mittal K, Nemirovsky A, Eremenko E, Vital A, Monovsky EIS, Chalifa-Caspi V, Friedman N, Yeger-Lotem E, Monsonego A. Aging promotes reorganization of the CD4 T cell landscape toward extreme regulatory and effector phenotypes. *Sci Adv* 2019; 5.

E7. Weatherly K, Bettonville M, Torres D, Kohler A, Goriely S, Braun MY. Functional profile of S100A4-deficient T cells. *Immun Inflamm Dis* 2015; 3: 431-444.

E8. Munier CML, van Bockel D, Bailey M, Ip S, Xu Y, Alcantara S, Liu SM, Denyer G, Kaplan W, group PS, Suzuki K, Croft N, Purcell A, Tscharke D, Cooper DA, Kent SJ, Zaunders JJ, Kelleher AD. The primary immune response to Vaccinia virus vaccination includes cells with a distinct cytotoxic effector CD4 T-cell phenotype. *Vaccine* 2016; 34: 5251-5261.

E9. Duggleby RC, Shaw TN, Jarvis LB, Kaur G, Gaston JS. CD27 expression discriminates between regulatory and non-regulatory cells after expansion of human peripheral blood CD4+ CD25+ cells. *Immunology* 2007; 121: 129-139.

E10. Pizzolato G, Kaminski H, Tosolini M, Franchini DM, Pont F, Martins F, Valle C, Labourdette D, Cadot S, Quillet-Mary A, Poupot M, Laurent C, Ysebaert L, Meraviglia S, Dieli F, Merville P, Milpied P, Dechanet-Merville J, Fournie JJ. Single-cell RNA sequencing unveils the shared and the distinct cytotoxic hallmarks of human TCRVdelta1 and TCRVdelta2 gammadelta T lymphocytes. *Proc Natl Acad Sci U S A* 2019; 116: 11906-11915.

E11. Park GH, Kim KY, Cheong JY, Cho SW, Kwack K. Association of GNLY genetic polymorphisms with chronic liver disease in a Korean population. *DNA Cell Biol* 2012; 31: 1492-1498.

E12. Turman MA, Yabe T, McSherry C, Bach FH, Houchins JP. Characterization of a novel gene (NKG7) on human chromosome 19 that is expressed in natural killer cells and T cells. *Hum Immunol* 1993; 36: 34-40.

E13. Sampath P, Moideen K, Ranganathan UD, Bethunaickan R. Monocyte Subsets: Phenotypes and Function in Tuberculosis Infection. *Front Immunol* 2018; 9: 1726.

E14. Ziegler-Heitbrock HW, Passlick B, Flieger D. The monoclonal antimonocyte antibody My4 stains B lymphocytes and two distinct monocyte subsets in human peripheral blood. *Hybridoma* 1988; 7: 521-527.

E15. Hruz T, Laule O, Szabo G, Wessendorp F, Bleuler S, Oertle L, Widmayer P, Gruissem W, Zimmermann P. Genevestigator v3: a reference expression database for the meta-analysis of transcriptomes. *Adv Bioinformatics* 2008; 2008: 420747.

E16. Heger L, Balk S, Luhr JJ, Heidkamp GF, Lehmann CHK, Hatscher L, Purbojo A, Hartmann A, Garcia-Martin F, Nishimura SI, Cesnjevar R, Nimmerjahn F, Dudziak D. CLEC10A Is a Specific Marker for Human CD1c(+) Dendritic Cells and Enhances Their Toll-Like Receptor 7/8-Induced Cytokine Secretion. *Front Immunol* 2018; 9: 744.

E17. Jahrsdorfer B, Vollmer A, Blackwell SE, Maier J, Sontheimer K, Beyer T, Mandel B, Lunov O, Tron K, Nienhaus GU, Simmet T, Debatin KM, Weiner GJ, Fabricius D. Granzyme B produced by human plasmacytoid dendritic cells suppresses T-cell expansion. *Blood* 2010; 115: 1156-1165.

E18. Masten BJ, Olson GK, Tarleton CA, Rund C, Schuyler M, Mehran R, Archibeque T, Lipscomb MF. Characterization of myeloid and plasmacytoid dendritic cells in human lung. *J Immunol* 2006; 177: 7784-7793.

E19. Aggarwal M, Villuendas R, Gomez G, Rodriguez-Pinilla SM, Sanchez-Beato M, Alvarez D, Martinez N, Rodriguez A, Castillo ME, Camacho FI, Montes-Moreno S, Garcia-Marco JA, Kimby E, Pisano DG, Piris MA. TCL1A expression delineates biological and clinical variability in B-cell lymphoma. *Mod Pathol* 2009; 22: 206-215.

E20. Chu PG, Arber DA. CD79: a review. *Appl Immunohistochem Mol Morphol* 2001; 9: 97-106.

E21. Lambert MP, Meng RH, Harper D, Xiao LQ, Marks MS, Poncz M. Megakaryocytes Exchange Significant Levels of Their Alpha-Granular PF4 with Their Environment. *Blood* 2014; 124.

E22. Laurenti E, Doulatov S, Zandi S, Plumb I, Chen J, April C, Fan JB, Dick JE. The transcriptional architecture of early human hematopoiesis identifies multilevel control of lymphoid commitment. *Nat Immunol*. 2013;14(7):756-63.

E23. Hafemeister C, Satija R. Normalization and variance stabilization of single-cell RNA-seq data using regularized negative binomial regression. *Genome Biology* 2019; 20.

E24. Kramer A, Green J, Pollard J, Jr., Tugendreich S. Causal analysis approaches in Ingenuity Pathway Analysis. *Bioinformatics* 2014; 30: 523-530.
